# Prediction of functional decline in community-dwelling older persons in general practice: a cohort study

**DOI:** 10.1186/s12877-018-0826-z

**Published:** 2018-06-11

**Authors:** Sophie C. E. van Blijswijk, Jeanet W. Blom, Anton J. M. de Craen, Wendy P. J. den Elzen, Jacobijn Gussekloo

**Affiliations:** 1 Department of Public Health and Primary Care, Leiden University Medical Center, PO Box 9600, 2300 RC Leiden, The Netherlands; 2 Department of Clinical Chemistry and Laboratory Medicine, Leiden University Medical Center, PO Box 9600, 2300 RC Leiden, The Netherlands; 3 Department of Gerontology and Geriatrics, Leiden University Medical Center, PO Box 9600, 2300 RC Leiden, The Netherlands

**Keywords:** Community-dwelling older person, General practice, Prediction, Functional decline, Readily available variables

## Abstract

**Background:**

A first step to offer community-dwelling older persons proactive care is to identify those at risk of functional decline within a year. This study investigates the predictive value of registered information, questionnaire and GP-opinion on functional decline.

**Methods:**

In this cohort study, embedded within the ISCOPE-trial, participants (≥75 years) completed the ISCOPE-screening questionnaire on four health domains. GPs gave their opinion on vulnerability of participants. Functional status was measured at baseline and 12 months (Groningen Activities Restriction Scale [GARS]). The outcome was functional decline (death, nursing home admission, 10% with greatest functional decline). The predictive value of pre-selected variables (age, sex, polypharmacy, multimorbidity, living situation; GPs’ opinion on vulnerability, number of domains with problems [ISCOPE-score]) was compared with the area under the curves (AUC) for logistic regression models.

**Results:**

2018 of the 2211 participants (median age 82.1 years [IQR 78.8–86.5], 68.0% female, median GARS 31 [IQR 24–41]) were visited at 12 months (median GARS 34 [IQR 26–44]). 394 participants (17.8%) had functional decline (148 died, 45 nursing home admissions, 201 with greatest functional decline). The AUC for age and sex was 0.602, increasing to 0.620 (*p* = 0.029) with polypharmacy, multimorbidity and living situation. The GPs’ opinion added more (AUC 0.672, *p* < 0.001) than the ISCOPE-score (AUC 0.649, *p* = 0.007). AUC with all variables was 0.686 (*p* = 0.016), and 0.643 for GPs’ opinion alone.

**Conclusions:**

The GPs’ opinion and ISCOPE-score improve this prediction model for functional decline based on readily available variables. GPs could identify older patients for further assessment with their clinical judgement.

**Trial registration:**

Netherlands trial register, NTR1946. Registered 10 August 2009.

**Electronic supplementary material:**

The online version of this article (10.1186/s12877-018-0826-z) contains supplementary material, which is available to authorized users.

## Background

It is important for older persons to be able to perform the basic activities of daily living (BADL) and instrumental activities of daily living (IADL). Previous research has shown that being independent has a positive effect on the quality of life [1, 2]. Therefore, many (inter)national healthcare programs aim to prevent and delay disability affecting independence, nursing home admission and mortality, with proactive healthcare [3–5]. Despite some promising results described by Beswick et al. [6], a more recent review shows that these programs often have limited effects [7].

A first and necessary step in proactive healthcare is to identify older persons at risk of functional decline and disability [8]. The disappointing effects of health care programs might be explained by inappropriate selection of the target population [9]. Some risk factors known to be associated with functional decline [10–14] and some predictors of dependency in BADL have been identified among specific populations [15, 16]. However, for community-dwelling older persons, the evidence to predict a decline in IADL and BADL remains limited and inconclusive. Even the value of several geriatric screening tools in prediction of functional decline appears to be limited [17]. More effective tools, both with a higher predictive value and easy to use in clinical practice, are needed. In the Netherlands, the general practitioner (GP) is closest to the patient and might be best able to identify those at risk of functional decline. Although some studies include possible predictors easily obtainable by the GP, the GPs’ clinical judgement and the interaction of health issues are less often taken into account [16, 18]. However, a recent study suggests the possible importance of the GPs’ judgement in predicting adverse outcomes [19]. The GPs’ judgement of functional status is a predictor that requires no additional investments in time and finances. It is therefore important to examine if the predictive value justifies to add this judgement to the diagnostic pathway for older patients.

Therefore, this study investigates whether the predictive value of several pre-selected clinical variables on functional decline which are readily available for the GP (age, sex, polypharmacy, multimorbidity and living situation) can be improved by adding the GPs’ opinion on the vulnerability of their patients and scores on the ISCOPE screening questionnaire on four health domains.

## Methods

### Study design and population

The present study is a longitudinal follow-up study embedded in the Integrated Systemic Care for Older People (ISCOPE) study. The ISCOPE Study was a large healthcare innovation trial conducted in the Netherlands [3].

Briefly, in ISCOPE, all eligible patients (≥75 years) in 59 general practices (*n* = 11,476) were invited (between September 2009 and September 2010) to participate. At baseline, they received the postal ISCOPE screening questionnaire (Additional file 1) with 23 questions on four health domains (somatic, functional, psychological and social). The ISCOPE-score is the number of domains with problems (i.e. when the participants experiences ≥2 items in this domain as a problem). GPs were asked to rate their opinion on the vulnerability of the participant on a three point scale (not vulnerable, possibly vulnerable, vulnerable). No guidelines were provided for interpretation of the term ‘vulnerable’ [20].

A selection of participants (15% with ISCOPE-score 0/1, 60% with ISCOPE-score 2, and 100% with ISCOPE-score 3/4) was visited at home by research nurses at baseline and at 12-month follow-up to collect more information on their functional status with the Minimal Data Set (MDS) questionnaire [21] and the Groningen Activities Restriction Scale (GARS) [22]. Participants were included in this analysis if data on their functional status and on all pre-selected variables were available at baseline and at 12 months, or if these data were available at baseline but the participant died or was admitted to a nursing home during the study period. All participants provided a written informed consent. For participants with cognitive problems this was provided by a representative.

### Pre-selected variables

As possible predictors we pre-selected variables known/or easy to obtain by the GP: age, sex, polypharmacy (> 3 medications), multimorbidity (> 1 of the following diseases: diabetes, stroke/cerebrovascular accident, myocardial infarction, heart failure, cancer, COPD/asthma, urine incontinence, osteoarthrosis hip/knee, osteoporosis, fracture, dizziness with falling, prostate symptoms, depression, anxiety disorder, dementia, hearing or visual problems), and living situation (home for older persons or independent, with or without others). All variables were self-reported in the ISCOPE screening questionnaire or the MDS questionnaire.

### Outcome measurement

Functional status was measured with the GARS (11 questions on BADL and 7 on IADL). Scores per question range from 1 point (I can do this fully independently, without any help) to 4 points (I can only do this with someone’s help). The total score ranges from 18 (completely independent) to 72 points (highly dependent) [22]. There has been no cut-off value defined for functional decline [23].

A low GARS at baseline can increase more than a high GARS at baseline (ceiling effect). Therefore we calculated the proportional increase in GARS from the potential increase in GARS from baseline ([GARS at 12 months – GARS at baseline] / [72 - GARS at baseline]) for each participant. Due to this correction, participants with the same increase in GARS at baseline, but a different potential increase in GARS, have a different proportional increase in GARS. Participants with the same GARS at baseline, and therefore the same potential increase in GARS, are comparable concerning their functional status. An increase in GARS within a group of participants with a comparable potential increase in GARS is likely to have a comparable impact on daily life. Therefore, we compared participants with other participants with the same potential increase in GARS. For this aim, the study population was divided into six categories according to their baseline GARS (18–26, 27–35, 36–44, 45–53, 54–62, 63–72). The number of participants per category could differ. It can be expected that the impact on daily life is larger for participants who have a larger proportional increase in GARS compared to other participants in their group with the same potential increase in GARS. Because of this, participants were considered to have a relevant functional decline when their proportional increase in GARS was higher than the proportional increase of 90% of the participants in their category. Those participants who were not visited after 12 months because they had died or were admitted to a nursing home, were also considered to have a relevant functional decline.

### Statistical methods

Patient characteristics are described as proportions, except for age and baseline GARS for which medians are reported. Baseline characteristics of individuals not included in the analysis were compared with those of the study participants.

**Fig. 1:**
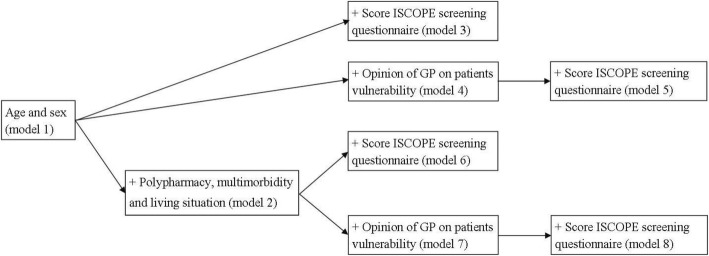
Steps in modelling of the ROC analyses

Associations between the pre-selected baseline variables and a relevant functional decline were tested with univariate logistic regression analysis. The different models were compared with the area under the curve (AUC) for the different receiver operating characteristics curves (ROC curves) in a step-wise manner. The ROC curve is a plot of the sensitivity against one minus the specificity for different cut-off points. The reference line is the line if the model has no discriminative power (AUC 0.5). The accuracy is poor with an AUC between 0.60 to 0.70, fair between 0.70 to 0.80, good between 0.80 to 0.90 and excellent between 0.90 to 1.0. The first model included age and sex. In separate analyses we added polypharmacy, multimorbidity and living situation (model 2), the ISCOPE-score (model 3), the GPs’ opinion on vulnerability (model 4), and the combination of the ISCOPE-score and the GPs’ opinion on vulnerability (model 5). In additional analyses we combined age, sex, polypharmacy, multimorbidity and living situation with either the ISCOPE-score (model 6), the GPs’ opinion on vulnerability (model 7), or both (model 8) (Fig. 1). The AUC for the GPs’ opinion alone is reported. Nagelkerkes’ R^2^ is reported for each model as well.

### Sensitivity analysis

After stratification into six groups based on the baseline GARS, additional ROC analyses were performed to check whether it was justified to combine these groups in the analysis.

As a sensitivity analysis we repeated the main ROC analyses but excluding those participants with a maximum baseline GARS-score of 72, since their score on the GARS could not increase.

All analyses were performed with SPSS 23.0 and STATA 12.0.

## Results

### Study population

**Fig. 2:**
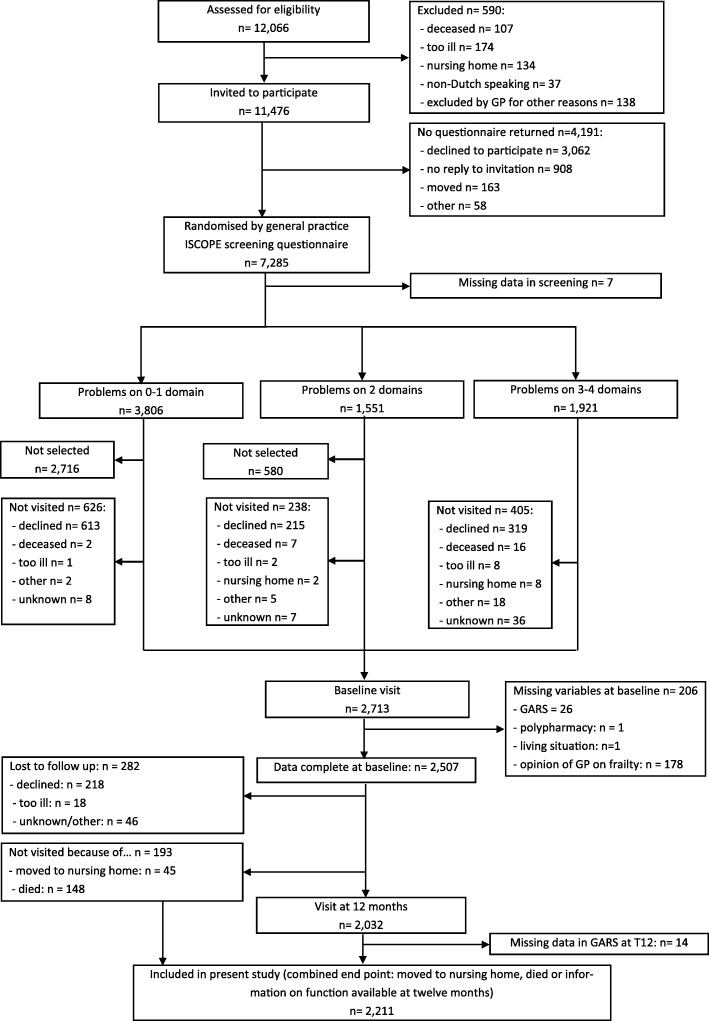
Flowchart of the study (*n* = 2211)

At baseline 7285 participants (response 63.5%) completed the ISCOPE screening questionnaire and 2713 (37.2%) were visited at home. Included in the present analysis (*n* = 2211) were participants with a GARS-score at baseline and at 12 months (*n* = 2018), those admitted to a nursing home (*n* = 45) and those who died (*n* = 148) (Fig. 2).

Median age was 82.1 (IQR 78.8–86.5) years, 68.0% was female and median GARS was 31 (IQR 24–41); 91% of the participants had multimorbidity and 67.9% polypharmacy. GPs considered 31.7% of the participants vulnerable and 40.4% not vulnerable. Also, 9.0% of the participants had an ISCOPE-score of zero and 16.9% had an ISCOPE-score of four (Table 1).

**Table 1:** Baseline characteristics of the study population (*n* = 2211) compared to baseline characteristics of participants not included in this study (*n* = 502)

	Study population *n* = 2211		Not included^a^*n* = 502		
Baseline characteristics	n	%	n	%	*p*-value^b^
Age at interview: years (median, IQR)	82.1 (78.8; 86.5)		82.6 (79.2; 87.3)		0.044
Baseline GARS (median, IQR)	31 (24; 41)		34 (25; 43)		0.006
Sex					
Male	707	32.0	150	29.9	0.362
Female	1504	68.0	352	70.1	
Polypharmacy			*n* = 501		
< 4 per day	710	32.1	185	36.9	0.039
4 or more per day	1501	67.9	316	63.1	
Multimorbidity					
Yes (> 1 chronic disease)	2011	91.0	458	91.2	0.843
No	200	9.0	44	8.8	
Living situation			*n* = 501		
Independent, alone/with others	1985	89.8	429	85.6	0.007
Home for older persons	226	10.2	72	14.4	
GP opinion on vulnerability			*n* = 323		
Not vulnerable	894	40.4	93	28.8	< 0.001
Possibly vulnerable	617	27.9	96	29.7	
Vulnerable	700	31.7	134	41.5	
ISCOPE-score on ISCOPE screening questionnaire					
0 (no domain with problems)	198	9.0	49	9.8	0.099
1 (1 domain with problems)	183	8.3	34	6.8	
2 (2 domains with problems)	614	27.8	119	23.7	
3 (3 domains with problems)	843	38.1	195	38.8	
4 (4 domains with problems)	373	16.9	105	20.9	

^a^Due to missing data not all variables add up to 502 participants

^b^Continuous data compared with Mann-Whitney test, percentages with Pearson’s chi-square test

At the 12-month follow-up, the GARS increased by 2 points (IQR − 1 to 6) to 34 (IQR 26 to 44) (*n* = 2018). The cut-off for relevant functional decline (≥ p90) per group was 19.7–100% of the potential increase (Additional file 2).

### Lost to follow-up and missing data

Participants were not included in this analyses when they were not visited at 12 months (i.e. refused further participation (*n* = 218), were too ill (*n* = 18), or for other/unknown reasons (*n* = 46)) or due to missing data at baseline (*n* = 206), or at 12 months (*n* = 14) (Fig. 2). In general, these participants were slightly older, had a higher baseline GARS, more often lived in a home for older persons, were more often vulnerable according to the GP, and had more often a higher ISCOPE-score (Table 1).

### Univariate association between pre-selected variables and functional decline

The 394 participants (17.8%) with relevant functional decline (148 died, 45 nursing home admissions, 201 with greatest functional decline) during follow-up, had at baseline a higher age, higher GARS, were less often female, more often had polypharmacy, more often lived in a home for older persons, were more vulnerable according to the GP, and had a higher ISCOPE-score. Not associated with functional decline was multimorbidity (Table 2).

**Table 2:** Baseline characteristics: comparison of participants without (*n* = 1817) and with (*n* = 394) a relevant functional decline (univariate logistic regression)

	Relevant functional decline					
	Without decline (*n* = 1817; 82.2%)		With decline (*n* = 394; 17.8%)			
Characteristics	n	%	n	%	Odds ratio	95% CI
Age in years (median, IQR)	81.8 (78.5–86.2)		83.5 (79.8–87.7)		1.06	1.04; 1.08
Baseline GARS (median, IQR)	30 (24–39)		36 (28–49)		1.04	1.03; 1.05
Sex						
Male	559	30.8	148	37.6	ref	
Female	1258	69.2	246	62.4	0.74	0.59; 0.93
Polypharmacy						
< 4 per day	603	33.2	107	27.2	ref	
4 or more per day	1214	66.8	287	72.8	1.33	1.05; 1.70
Multimorbidity						
No	169	9.3	31	7.9	ref	
Yes (> 1 chronic disease)	1648	90.7	363	92.1	1.20	0.81; 1.79
Living situation						
Independent, alone/with others	1656	91.1	329	83.5	ref	
Home for older persons	161	8.9	65	16.5	2.03	1.49; 2.77
GP opinion on vulnerability						
Not vulnerable	802	44.1	92	23.4	ref	
Possibly vulnerable	518	28.5	99	25.1	1.67	1.23; 2.26
Vulnerable	497	27.4	203	51.5	3.56	2.72; 4.67
ISCOPE-score on ISCOPE screening questionnaire						
0 (no domain with problems)	179	9.9	19	4.8	ref	
1 (1 domain with problems)	172	9.5	11	2.8	0.60	0.28; 1.30
2 (2 domains with problems)	508	28.0	106	26.9	1.97	1.17; 3.30
3 (3 domains with problems)	687	37.8	156	39.6	2.14	1.29; 3.54
4 (4 domains with problems)	271	14.9	102	25.9	3.55	2.10; 5.99

### Predictive value of pre-selected variables on a functional decline

**Fig. 3:**
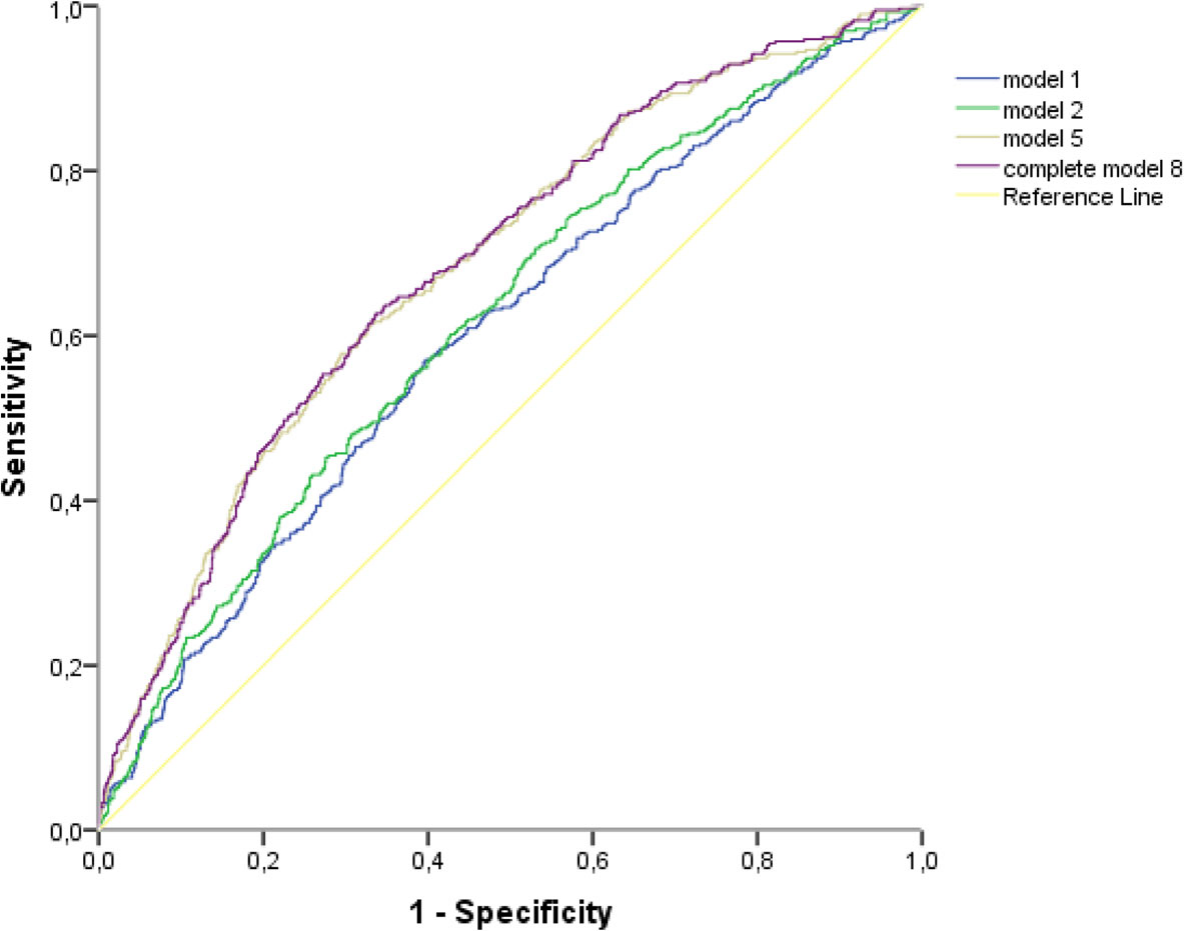
ROC curve of the different models predicting a relevant decline in functional status (*n* = 2211). Model 1: age and sex; Model 2: age, sex, polypharmacy, multimorbidity and living situation; Model 5: age, sex, GP opinion and ISCOPE-score; Model 8: age, sex, polypharmacy, multimorbidity, living situation, GP opinion and ISCOPE-score

The AUC for age and sex was 0.602 (model 1). The AUC increased to 0.620 (*p* = 0.0 29, compared to model 1) when polypharmacy, multimorbidity and living situation were added (model 2), to 0.644 (*p* < 0.001, compared to model 1) with addition of the ISCOPE-score (model 3), and to 0.669 (*p* < 0.001, compared to model 1) with addition of GPs’ opinion on vulnerability (model 4). With a combination of age, sex, ISCOPE-score and GPs’ opinion (model 5), the AUC increased from 0.669 to 0.684 (*p* = 0.009, compared to model 4). The predictive value of the more extensive model with age, sex, polypharmacy, multimorbidity and living situation increased more with the GPs’ opinion on vulnerability (model 7: AUC 0.672, *p* < 0.001, compared to model 2) than with the ISCOPE-score (model 6: AUC 0.649, *p* = 0.007, compared to model 2). With all variables included (model 8), the AUC increased from 0.672 to 0.686 (*p* = 0.016, compared to model 7) (Table 3; Fig. 3). The AUC for the GPs’ opinion alone was 0.643.

**Table 3:** Multivariate models to predict a relevant decline of functional status (*n* = 2211)

Variables included in the model		AUC	compared to	delta AUC	*p*-value	R^2a^
Model 1	Age and sex	0.602				0.033
Model 2	model 1, polypharmacy, multimorbidity and living situation	0.620	model 1	0.018	0.029	0.042
Model 3	model 1 and ISCOPE-score	0.644	model 1	0.024	< 0.001	0.066
Model 4	model 1 and GP opinion on vulnerability	0.669	model 1	0.049	< 0.001	0.086
Model 5	model 1, ISCOPE-score and GP opinion on vulnerability	0.684	model 4	0.015	0.009	0.102
Model 6	model 2 and ISCOPE-score	0.649	model 2	0.029	0.007	0.069
Model 7	model 2 and GP opinion on vulnerability	0.672	model 2	0.052	< 0.001	0.090
Model 8	model 2, ISCOPE-score and GP opinion on vulnerability	0.686	model 7	0.014	0.016	0.105

^a^Nagelkerke

### Sensitivity analysis

After stratification of the population into six groups according to the baseline GARS (*n* = 60–735), a similar trend in the AUC was observed in the different group (Additional file 2).

At baseline, 7 participants had a maximum GARS-score of 72 which was the same at the 12-month follow-up; after excluding these participants from the analysis, the results did not change (data not shown).

## Discussion

The predictive value of a model for functional decline using variables readily available for the GP (age, sex, polypharmacy, multimorbidity and living situation) improves when the ISCOPE-score or the GPs’ opinion on vulnerability is added to the model. The GPs’ opinion alone has a predictive value of 0.643. Data readily available for the GP can be used in combination with the GPs’ opinion to predict functional decline. If the GP is not familiar with the patient, the ISCOPE-score can be used instead of the GPs’ opinion with only a small loss of discriminative power.

### Explanation of findings

This study is in line with earlier research that shows that geriatric screening tools have limited use in predicting functional decline [17]. It builds on previous work from Van Kempen et al. who published a two-step tool (Easycare TOS) to identify older persons at risk for negative outcomes after 1 year. The tool uses variables easy to obtain in clinical practice in addition to the GPs’ opinion on frailty [16]. In contrast with the present study a decline in IADL was not considered to be a negative outcome.

Also a small study from the Netherlands by Sutorius et al. suggests that the GP predicts adverse outcomes (6 year-mortality or long term care admission) better than several other methods to identify frail older persons in primary care [19]. Although the results could have been influenced since only one GP judged the patients [20], this interesting finding supports the idea of the relevance of the GPs’ judgement of the patient.

Research on this topic has its challenges because there is no consensus on the definition of disability and functional decline [24]. Most studies on the prediction of functional decline use only a decline in BADL as outcome [16] and sometimes include scores on IADL as possible predictors [12, 25]. However, a decline in IADL status is also relevant for older participants because it has implications for their independence. Anderson et al. and van Houwelingen et al. include IADL disability. They describe disability as an unstable state that can improve or worsen over time and they define categories for disability, i.e. independent/no disability, disability in IADL, (mild) disability in BADL, and institutionalisation or death [11, 13]. Although these categories include IADL disability, small changes in IADL or BADL performance are difficult to detect. Therefore, in this study we combined BADL and IADL in the primary outcome. The definition used for a relevant functional decline is rather complex but with this definition we compare participants to other participants with the same functional status at baseline, taking their own functional status at baseline into account.

### Strengths and limitations

The strengths of this large cohort study with participants aged ≥75 years are that: 1) we included only those variables known by the GP combined with the GPs’ clinical judgement and the ISCOPE-score, to develop a prediction model feasible for clinical practice; 2) we used a decline in BADL *and* IADL as the primary outcome since both contribute to self-reliance and independence; and 3) relevant patient characteristics and prospective data on functional status were available for a large sample of community-dwelling older participants.

Some limitations also need addressing: 1) GPs were asked their opinion on patients’ vulnerability at baseline. Earlier research showed that GPs take somatic and psychological characteristics into account, but weigh the functional and social characteristics differently when assessing vulnerability [20]; 2) a follow-up period > 12 months with repeated measurements may be needed to reveal more subtle changes in functional status; 3) reasons for drop-out may have been related to the outcome of the study. Since participants that dropped-out were slightly older, had a higher baseline GARS-score, lived more often in a home for older persons, were more vulnerable according to the GP and had a higher ISCOPE-score, the true predictive value may be higher than we observed.

### Implications for research and practice

To deliver proactive care in general practice, efficient identification of older persons at risk for functional decline is important [8]. It appears that the prediction of functional decline by readily available variables can be improved by adding the GPs’ opinion, or the ISCOPE-score when the GP is not familiar with the patient. This knowledge might be useful to select participants for a more extensive evaluation.

To improve the prediction of functional decline and identification of older persons at risk, a relevant outcome measure needs to be defined: e.g., a relevant cut-off for the measurement tools for functional decline, a definition in terms of disability transitions [11, 13], or a more personalised outcome measurement as goal attainment scaling [26]. Also, other factors such as unexpected adverse health events (e.g. hip fracture, hospital admissions, or the loss of a spouse or primary caregiver) could be important predictors. Furthermore, we agree with Sutorius et al. [19] that identification of older persons at risk for a functional decline it only a first step. Second should be establishing which older persons at risk, are likely to benefit from pro-active care to prevent a functional decline. Research on these topics might offer new opportunities for prevention of disability and dependence.

## Conclusions

The predictive value of a model for functional decline using variables readily available for the GP (age, sex, polypharmacy, multimorbidity and living situation) improves when the ISCOPE-score or the GPs’ opinion on vulnerability is added to the model. The GPs’ opinion alone has a predictive value of 0.643. Until it becomes possible to predict a risk of functional decline more accurately, it seems beneficial to use the clinical judgement of GPs to select older persons in probable need of more extensive assessment.

## Additional files

Additional file 1: ISCOPE screening questionnaire [3]. (pdf 64 kb)

Additional file 2: Sensitivity analysis: multivariate models to predict a relevant decline in functional status stratified by baseline GARS (*n* = 2211) and change in GARS, and number of participants stratified by baseline GARS (*n* = 6). (pdf 402 kb)

## Abbreviations

AUC: Area under the curve
BADL: Basic activities of daily living
COPD: Chronic obstructive pulmonary disease
GARS: Groningen Activities Restriction Scale
GP: General practitioner
IADL: Instrumental activities of daily living
IQR: Interquartile range
ISCOPE study: Integrated systemic care for older people study
MDS: Minimal data set
ROC curve: Receiver operating characteristics curves
