# Supplementary material for: Prediction of functional decline in community-dwelling older persons in general practice: a cohort study
Source: BMC Geriatr. 2018 Jun 11;18:140. doi: 10.1186/s12877-018-0826-z (PMC6001140; doi:10.1186/s12877-018-0826-z)
Supplement: Supplementary file 1 — ISCOPE screening questionnaire [3]. (pdf 64 kb) [file 12877_2018_826_MOESM1_ESM.pdf]

Additional file 1: Appendix 1. ISCOPE screening questionnaire [3]

Daily life abilities
These first questions relate to how you function/manage day-to-day life.
You may be helped in these activities by aids such as a stick walking frame or wheelchair.

1. Can you do the shopping without help from anyone else?
Yes / No

2. Can you walk outdoors without help from anyone else?
Yes / No

3. Can you dress and undress yourself without help from anyone else?
Yes / No

4. Can you go to the toilet without help from anyone else?
Yes / No

5. Can you manage your finances yourself (collect your money, pay your bills)?
Yes / No

6. How well would you say you cope with your general day-to-day life?
Well / Average /Not at all well

Health and illness

7. Which mark would you give for your physical fitness?
1 2 3 4 5 6 7 8 9 10
Not at all fit *Very fit*

8. Do you experience day-to-day problems due to poor eyesight (even if you wear glasses or contact lenses)?
Yes / No

9. Do you experience day-to-day problems due to poor hearing (even if you wear a hearing aid)?
Yes / No

10. Do you experience problems with incontinence of urine or stool?
Yes / No

11. Do you experience daily problems due to pain?
Yes / No

12. Have you lost weight (more than 6 kg) in the last 6 months unintentionally?
Yes / No

13. Are you using more than 4 different kinds of medicine at the moment?
Yes / No

14. Have you had a fall in the last month?
Yes / No

15. Have you been admitted to the hospital in the last 6 months?
Yes / No

Psychological functioning

16. Do you feel you have memory complaints?
Yes /Sometimes/ No

17. Have you recently felt sad or depressed?
Yes /Sometimes/ No

18. Have you recently felt nervous or anxious?
Yes /Sometimes/ No

19. Do you feel pretty worthless at the moment?
Yes /Sometimes/ No

Social functioning

20. Do you feel that your life is empty?
Yes /Sometimes/ No

21. Do you feel the lack of a close friend?
Yes /Sometimes/ No

22. Do you feel left alone sometimes?
Yes /Sometimes/ No

23. Do you feel there are enough people with whom you feel a close connection?
Yes /Sometimes/ No

24. Do you receive help from anybody in you immediate surrounding because you are unable to do things for yourself?
Yes / No

25. Has anyone helped you to fill in this questionnaire?
No, I have filled in the questionnaire myself.
Yes, someone helped me to answer these questions.
Somebody has answered them for me.

26. At the moment, which health complaints limit you the most in your day-to-day life?
