# Supplementary material for: Prediction of functional decline in community-dwelling older persons in general practice: a cohort study
Source: BMC Geriatr. 2018 Jun 11;18:140. doi: 10.1186/s12877-018-0826-z (PMC6001140; doi:10.1186/s12877-018-0826-z)
Supplement: Supplementary file 2 — Sensitivity analysis: multivariate models to predict a relevant decline in functional status stratified by baseline GARS (n = 2211) and change in GARS, and number of participants stratified by baseline GARS (n = 6). (pdf 402 kb) [file 12877_2018_826_MOESM2_ESM.pdf]

Additional file 2: Appendix 2. Multivariate models to predict a relevant decline in functional status stratified by baseline GARS (n=2211)

	**group 1[a] (n=735)**			**group 2[a] (n=647)**			**group 3[a] (n=418)**			**group 4[a] (n=241)**			**group 5[a] (n=110)**			**group 6[a] (n=60)**		
	AUC	delta AUC	p-value	AUC	delta AUC	p-value	AUC	delta AUC	p-value	AUC	delta AUC	p-value	AUC	delta AUC	p-value	AUC	delta AUC	p-value
Model 1	0,602			0,584			0,540			0,627			0,638			0,702		
Model 2	0,635	0,033	0.1651	0,586	0,002	0.8619	0,577	0,037	0.2483	0,648	0,021	0.2295	0,662	0,024	0.4783	0,709	0,007	0.8349
Model 3	0,654	0,052	0.0353	0,632	0,048	0.0913	0,578	0,038	0.2483	0,635	0,008	0.6455	0,675	0,037	0.4056	0,721	0,019	0.6383
Model 4	0,637	0,035	0.1773	0,659	0,075	0.0172	0,602	0,062	0.1035	0,693	0,066	0.0329	0,664	0,026	0.4887	0,750	0,048	0.3224
Model 5	0,671	0,034	0.1119	0,680	0,021	0.1530	0,628	0,026	0.1077	0,700	0,007	0.5683	0,706	0,042	0.2356	0,773	0,023	0.4913
Model 6	0,668	0,033	0.0801	0,628	0,042	0.1144	0,613	0,036	0.1906	0,668	0,020	0.2236	0,698	0,036	0.3880	0,739	0,030	0.3183
Model 7	0,653	0,018	0.3596	0,658	0,072	0.0232	0,633	0,056	0.0874	0,701	0,053	0.0720	0,688	0,026	0.4738	0,766	0,057	0.1813
Model 8	0,682	0,029	0.1108	0,681	0,023	0.1053	0,653	0,020	0.2058	0,715	0,014	0.2915	0,726	0,038	0.2512	0,775	0,009	0.7462

Models | 1: Age and sex | 2: model 1 + polypharmacy, multimorbidity and living situation | 3: model 1 + ISCOPE-score | 4: model 1 + opinion of GP | 5: model 1 + ISCOPE-score + opinion of GP | 6: model 2 + ISCOPE-score | 7: model 2 + opinion of GP | 8: model 2 + ISCOPE-score + opinion of GP||

[a]Change in GARS and number of participants stratified by baseline GARS (n=6)

Group No	Baseline GARS	Participants with valid delta GARS n	delta GARS in 12 months Median (IQR)	p90 decline in functional status[#]	participants died/ moved to NH n	total participants n
1	18-26	717	2 (0; 5)	0.1969	18	735
2	27-35	607	2 (-1; 7)	0.2720	40	647
3	36-45	377	3 (-1; 8)	0.3939	41	418
4	45-53	203	1 (-2; 5)	0.4703	38	241
5	54-62	74	1 (-3.35; 4)	0.5359	36	110
6	63-72	40	0 (-2; 2)	1	20	60

[#]p90 of score calculated with (GARS at 12 months – GARS at baseline) / (72-GARS at baseline)
